# First description of the male and hemipenial morphology of *Opisthotropis haihaensis* Ziegler et al., 2019 (Serpentes, Natricidae), with updated diagnosis and distribution

**DOI:** 10.3897/BDJ.13.e167521

**Published:** 2025-11-20

**Authors:** Tierui Zhang, Jinlong Ren, Maoliang Li, Yuhao Xu, Xinge Wang, Nikolay A. Poyarkov, Tan Van Nguyen, Song Huang

**Affiliations:** 1 The Anhui Provincial Key Laboratory of Biodiversity Conservation and Ecological Security in the Yangtze River Basin, College of Life Sciences, Anhui Normal University, Wuhu, China The Anhui Provincial Key Laboratory of Biodiversity Conservation and Ecological Security in the Yangtze River Basin, College of Life Sciences, Anhui Normal University Wuhu China; 2 CAS Key Laboratory of Mountain Ecological Restoration and Bioresource Utilization, Ecological Restoration and Biodiversity Conservation Key Laboratory of Sichuan Province, Chengdu Institute of Biology, Chinese Academy of Sciences, Chengdu, China CAS Key Laboratory of Mountain Ecological Restoration and Bioresource Utilization, Ecological Restoration and Biodiversity Conservation Key Laboratory of Sichuan Province, Chengdu Institute of Biology, Chinese Academy of Sciences Chengdu China; 3 State Key Laboratory of Plateau Ecology and Agriculture, Xining, China State Key Laboratory of Plateau Ecology and Agriculture Xining China; 4 Department of Vertebrate Zoology, Lomonosov Moscow State University, Moscow, Russia Department of Vertebrate Zoology, Lomonosov Moscow State University Moscow Russia; 5 The School of Medicine & Pharmacy, Duy Tan University, Da Nang, Vietnam The School of Medicine & Pharmacy, Duy Tan University Da Nang Vietnam; 6 Center for Entomology & Parasitology Research, Duy Tan University, Da Nang, Vietnam Center for Entomology & Parasitology Research, Duy Tan University Da Nang Vietnam

**Keywords:** Natricidae, Opisthotropis, sexual dimorphism, subtropical moist broadleaf forest

## Abstract

**Background:**

The Haiha Mountain Stream Keelback, *Opisthotropis haihaensis* Ziegler, Pham, Nguyen, Nguyen, Wang, Wang, Stuart & Le, 2019, was originally described based on a single female specimen from northern Vietnam. To date, only two female specimens have been reported, rendering *O. haihaensis* one of the least studied members of its genus.

**New information:**

During recent herpetological surveys in Guangxi Zhuang Autonomous Region, southern China, one male and one female specimen of *Opisthotropis* were collected from Qinzhou City. Phylogenetic analyses and detailed morphological comparisons identified these individuals as *O. haihaensis*. Based on these new specimens, we provide the first description of the male of this species, including hemipenial morphology, along with revised diagnostic features and new data on its natural history and distribution.

## Introduction

The genus *Opisthotropis* Günther, 1872 (Natricinae) comprises stream-dwelling snakes distributed from southern China and the Indochina Peninsula to the Ryukyu Archipelago, Sumatra, and the Philippines (Poyarkov et al. 2023, Uetz et al. 2025). Currently, 25 species are recognized in the genus, including nine described within the past two decades (Teynié et al. 2014, Wang et al. 2017, Wang et al. 2017, Ren et al. 2017, Ziegler et al. 2019, Wang et al. 2020). Owing to their aquatic and cryptic lifestyles, many *Opisthotropis* species remain poorly known and are often represented solely by their type series (Stuart and Chuaynkern 2007, Teynié et al. 2014, Wang et al. 2020, Gao et al. 2024).

One such example is *Opisthotropis haihaensis* Ziegler, Pham, Nguyen, Nguyen, Wang, Wang, Stuart & Le, a member of the *O. maculosa* complex together with *O. maculosa* Stuart & Chuaynkern and *O. hungtai* Wang, Lyu, Zeng, Lin, Yang, Nguyen, Le, Ziegler & Wang. Species within this complex share the following combination of features: a single prefrontal scale; dorsal scale rows in 15–15–15; smooth dorsal scales; a dark brown to blackish dorsum on the body and tail, with each scale bearing a pale yellow spot; yellow chin shields with dark mottling; and yellow ventral surfaces with dark brown lateral margins and scattered brown flecks (Stuart and Chuaynkern 2007, Yang et al. 2011, Ziegler et al. 2018, Wang et al. 2020).

The *maculosa* complex was first recorded in China by Yang et al. (2011), based on specimens from Guangdong Province and Guangxi Zhuang Autonomous Region (ZAR). Subsequently, Ziegler et al. (2019) described *O. haihaensis* as a distinct species based on a single female from Tai Chi Village, Quang Son Commune, Hai Ha District, Quang Ninh Province, northern Vietnam. This specimen had previously been misidentified as *O. maculosa* sensu Stuart & Chuaynkern, which was originally described from Phu Wua Wildlife Sanctuary, Boong Klar District, Nong Khai Province, Thailand (Nguyen et al. 2018). Later, Wang et al. (2020) re-examined the Chinese records assigned to *O. maculosa* by Yang et al. (2011), describing *O. hungtai* as a new species from Guangdong Province and Guangxi ZAR. They also confirmed the presence of *O. haihaensis* in China for the first time, based on an adult female specimen from Shiwandashan National Nature Reserve, Fangchenggang District, Guangxi ZAR, and recommended the removal of *O. maculosa* from the Chinese herpetofauna.

Based on two female specimens, *O. haihaensis* was initially diagnosed by the following combination of characters: total length 500–509 mm in adult females; relatively long tail (tail length/total length ratio 0.22); internasal not in contact with loreal; prefrontal not contacting supraocular; frontal contacting preocular; one preocular and one or two postoculars; temporals 1+1; eight supralabials, with the 4^th^ and 5^th^ entering the orbit; 22–24 maxillary teeth; anterior chin shields longer than posterior; 164–169 ventrals (plus two preventrals); 75–79 subcaudals; nasal cleft directed towards first supralabial; dorsal scales in 15–15–15 rows, smooth; tail scales smooth or indistinctly keeled; chin shields yellow with dark mottling; and body and tail dorsum dark, each scale with a pale yellow spot (Ziegler et al. 2019, Wang et al. 2020). Despite its recent description, the lack of male specimens has hindered a comprehensive understanding of the species’ diagnostic characters and distributional limits.

During a recent survey in Qinbei District, Qinzhou City, Guangxi ZAR, China, two specimens (one male and one female) referable to *Opisthotropis* were collected. Morphological comparisons and phylogenetic analyses confirmed their identity as *O. haihaensis*. In this study, we provide the first detailed morphological description of the male, including hemipenial morphology, update the species’ known distribution, and revise its diagnosis based on new material.

## Materials and methods

### Sampling

Two specimens of *Opisthotropis* were collected from Qinbei District, Qinzhou City, Guangxi ZAR, China. These specimens were humanely euthanized with 0.7% tricaine methanesulfonate (MS222) solution. Fresh liver tissue was extracted and immediately preserved in 95% ethanol. Specimens were preserved in 75% ethanol for permanent storage, and deposited in Anhui Normal University Museum (ANU). Sampling procedures involving live snakes were approved by the Animal Ethics Committee of Anhui Normal University and complied with the Wild Animals Protection Law of China.

### Phylogenetic analyses

Total genomic DNA was extracted from preserved liver tissue with OMEGA Tissue DNA Kit D3396 (Omega Bio-Tek, Norcross, GA, USA). A fragment of the mitochondrial cytochrome *b* (Cyt *b*) gene was amplified using the primer pair L14910 (5’-GACCTGTGATMTGAAACCAYCGTTGT-3’) and H16064 (5’-CTTTGGTTTACAAGAACAATGCTTTA-3’) (Burbrink et al. 2000). The double-stranded products were sequenced by Sangon Biotech (Shanghai, China), and raw sequences were assembled using SeqMan in the DNASTAR software package (Burland 2000). The sequences obtained from this study were uploaded to DNA Data Bank of Japan (DDBJ).

Following Gao et al. (2024), 34 sequences from 18 known *Opisthtropis* species and three out-group species, *Hebius johannis* (Boulenger), *Natrix natrix* (Linnaeus) and *Rhabdophis leonardi* (Wall) were obtained from GenBank and incorporated into our dataset (Table 1). DNA sequences were aligned by the Clustal W algorithm with default parameters (Thompson et al. 1997) and trimmed with gaps partially deleted in MEGA X (Kumar et al. 2018). Bayesian inferences (BI) was conducted in MRBAYES v. 3.2.7a (Ronquist et al. 2012) under the GTR + I + G model on Phylosuite v1.2.3 (Zhang et al. 2020, Xiang et al. 2023). In the BI analysis, three independent runs were conducted with 1 × 10^7^ generations and sampled every 1000 generations with the first 25% samples were discarded as burn-in. In the ML analysis, the bootstrap consensus tree was inferred from 1000 replicates. Maximum likelihood (ML) was conducted under the best-fit substitution model (GTR + I + G) in RaxmlGUI 1.3 (Silvestro and Michalak 2012). Bootstrap proportions (BSP) were investigated with 1,000 bootstrap replicates using the fast-bootstrapping algorithm. Uncorrected pairwise genetic distances (p-distance) of Cyt *b* gene among congeners were calculated with MEGA X (Kumar et al. 2018).

### Morphological examination

Morphological descriptions followed Ziegler et al. (2019), Wang et al. (2020) and Gao et al. (2024). Body measurements and their abbreviations are as follows: eye horizontal diameter (ED), head length (HL), maximum head width (HW), tail length (TaL), total length (TL), snout length (SnL), snout width (SnW), interorbital distance (IOD), distance between the lower margins of eye and of lip (SoL). All measurements, except for tail length and total length, were obtained with a digital slide-caliper to the nearest 0.01 mm.

Scalation features and their abbreviations are as follows: dorsal scale row counts (DSR), supralabial counts (SL), infralabial counts (IL), internasal counts (IN), chin shield counts (CS), frontal counts (F), parietal counts (P), preocular counts (PrO), postocular counts (PtO), ventral counts (VEN), subcaudal counts (SC), prefrontal in contact with supraocular or not (PrF-SpO), preocular in contact with frontal (PrO-F), loreal entering orbit (L-orbit), internasals in contact with loreal (IN-L), supralabials in contact with orbit (SL-orbit), infralabials in contact with anterior chin shields (IL-aCS), temporal counts (TEM), cloacal plate entire or divided (CP), dorsal scale surface of tail (DST). The number of ventral scales was counted according to Dowling (1951). Symmetric characters were given as left/right and averages were used in the analyses.

Gender of specimen was determined through dissection. The hemipenes of the male specimen was everted from the left side for the hemipenial description. The preparation and measurement method of hemipenes followed Ren et al. (2022). The everted hemipenes were re-inflated with coloured petroleum jelly and then preserved in 75% ethanol. Photographs of the hemipenes were taken using digital camera attached to a tripod head and multifocal photographs were combined and montaged using the Helicon Focus (7.0.2 Pro) software. Hemipenial morphology and terminology followed Dowling and Savage (1960), Zhang et al. (1984) and Ren et al. (2022).

Museum and collection abbreviations are as follows: ANU = Anhui Normal University, Wuhu, China; FMNH = Field Museum of Natural History, Chicago, USA; IEBR = Institute of Ecology and Biological Resources, Vietnam Academy of Science and Technology, Hanoi, Vietnam; KFBG = Herpetological Collection of Kadoorie Farm and Botanic Garden, Hong Kong, China; SYS = Sun Yat-sen University, Guangzhou, Guangdong, China.

## Taxon treatments

### Opisthotropis haihaensis

Ziegler, Pham, Nguyen, Nguyen, Wang, Wang, Stuart & Le, 2019

3836EA3B-2B5D-57A3-B940-9DDBF97141C6

#### Materials

- **Type status:** Other material. **Occurrence:** catalogNumber: ANU 20240076; individualCount: 1; sex: male; lifeStage: adult; occurrenceID: 37B587F0-02CC-5439-B859-E7923E8C247A; **Taxon:** taxonID: urn:lsid:biosci.ohio-state.edu:osuc_names:275502; scientificName: *Opisthotropis haihaensis*; class: Reptilia; order: Squamata; family: Natricidae; genus: Opisthotropis; specificEpithet: *haihaensis*; scientificNameAuthorship: Ziegler, Pham, Nguyen, Nguyen, Wang, Wang, Stuart & Le, 2019; **Location:** country: China; countryCode: CN; stateProvince: Guangxi; county: Qinbei; municipality: Qinzhou; locality: Tianmushan National Nature Reserve, Mt. Xianrending; verbatimLocality: 570 m; verbatimElevation: 1200 m; verbatimLatitude: 22.006365°N; verbatimLongitude: 108.206833°E; verbatimCoordinateSystem: WGS84; **Event:** eventDate: 25-Jun-24; eventRemarks: collected by Tierui Zhang; **Record Level:** language: en; collectionCode: Reptilia; basisOfRecord: PreservedSpecimen
- **Type status:** Other material. **Occurrence:** catalogNumber: ANU 20240077; individualCount: 1; sex: female; lifeStage: adult; occurrenceID: AAD9F7D6-CE03-591A-AD77-98734F6D0E95; **Taxon:** taxonID: urn:lsid:biosci.ohio-state.edu:osuc_names:275502; scientificName: *Opisthotropis haihaensis*; class: Reptilia; order: Squamata; family: Natricidae; genus: Opisthotropis; specificEpithet: *haihaensis*; scientificNameAuthorship: Ziegler, Pham, Nguyen, Nguyen, Wang, Wang, Stuart & Le, 2019; **Location:** country: China; countryCode: CN; stateProvince: Guangxi; county: Qinbei; municipality: Qinzhou; locality: Tianmushan National Nature Reserve, Mt. Xianrending; verbatimLocality: 570 m; verbatimElevation: 1200 m; verbatimLatitude: 22.006365°N; verbatimLongitude: 108.206833°E; verbatimCoordinateSystem: WGS84; **Event:** eventDate: 25-Jun-24; eventRemarks: collected by Tierui Zhang; **Record Level:** language: en; collectionCode: Reptilia; basisOfRecord: PreservedSpecimen

#### Description of male specimen ANU 20240076

**Measurements and scalation.** Body slender and cylindrical (TL 428.0 mm); head short and broad, dorsally depressed, barely distinct from neck (HL/HW 1.87); snout moderate (SnL/SnW 0.74); eyes in medium size (ED/SoL 0.97); pupil round; interorbital distance large (IOD/HW 0.66); minute granular asperities absent on head scales; tail relatively long, tapering posteriorly (TaL 113.9 mm, TaL/TL 0.27); nostril oval-shaped, locating in the upper middle part of nasal, directed upwards.

Dorsal scales in 15 rows throughout the body; the outermost rows of dorsal scales slightly enlarged, while the rest ones homogeneous in size, smooth throughout; vertebral scales not enlarged; preventrals 1, ventrals 168; cloacal plate divided; subcaudals 87 paired, with single terminal rigid tip, smooth entirely.

Rostral crescent-shaped, wider than high, visible from above; nasals large, wider than high, divided below nostril by a distinct furrow; nasals in contact with the first two supralabials, rostral, internasal, prefrontal and loreal; internasals paired, trapezoid, longer than wide, curved outward posteriorly, in contact with rostral, nasal, and prefrontal; prefrontal single, much broader than long, anteriorly pointed, in contact with internasals, nasals, loreals, preoculars, and frontal; frontal single, pentagonal or near equilateral triangle, almost equal in width and length, tapering posteriorly, equal to its distance from tip of snout; parietals nearly 1.8 times as long as wide, parietal sutures nearly equal to frontal in length; 1/1 supraocular, distinctly longer than wide, not in contact with prefrontal; 1/1 loreal, pentagonal, wider than high, not entering orbit, surrounded by second and third supralabials, nasal, prefrontal and preocular; 1/1 preocular, large, hexagonal, higher than wide, reaching frontal; subocular absent; 2/2 postocular, upper pair is almost twice the length and width of lower pair; 7/7 supralabials, 1^st^–5^th^ higher than wide, only 5^th^ entering orbit on left lateral, 4^th^ and 5^th^ entering orbit on right lateral (4-1-2/3-2-2), 7^th^ largest; 1+1/1+1 temporals, anterior one very long and narrow, in broad contact with 5^th^–7^th^ supralabials and parietals, posterior one more strongly developed; infralabials 8/8, first pair in contact behind small mental, 1^st^–5^th^/ 1^st^–5^th^ in contact with anterior chin shields, 5^th^ infralabial largest; anterior chin shields longer than posterior ones, posterior chin shields do not contact with each other.

**Dentition.** Maxillary teeth 22, subequal, densely set, without diastema.

**Figure 1.**
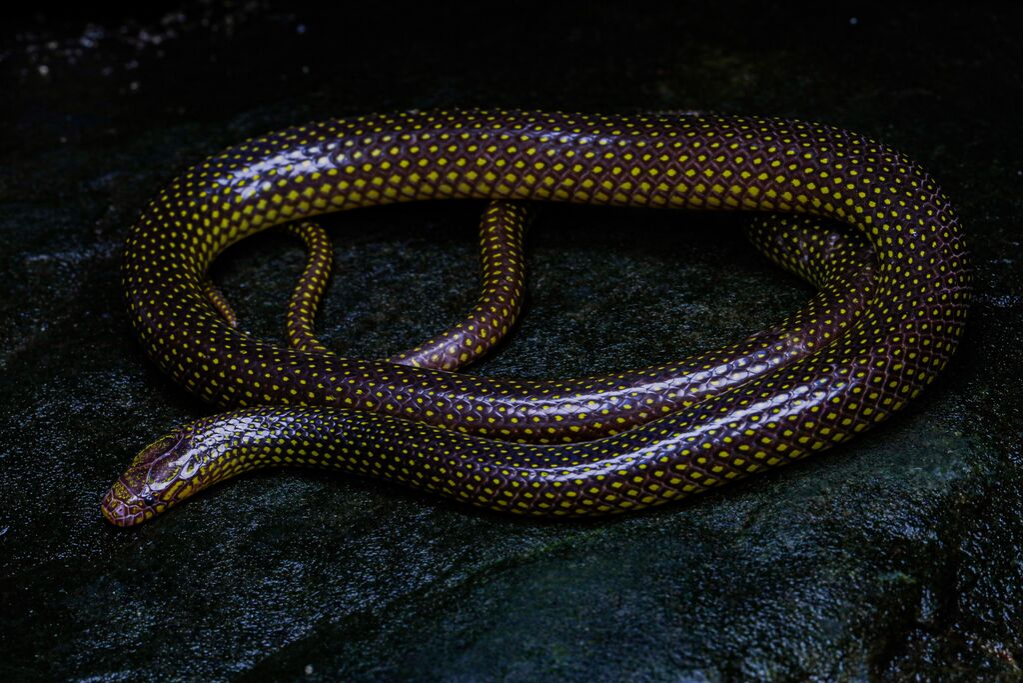
Live photographs of *Opisthotropis haihaensis* (specimen ANU 20240076, male) in situ from Qinzhou, Guangxi, China. Photographed by T.R. Zhang.

**Figure 2.**
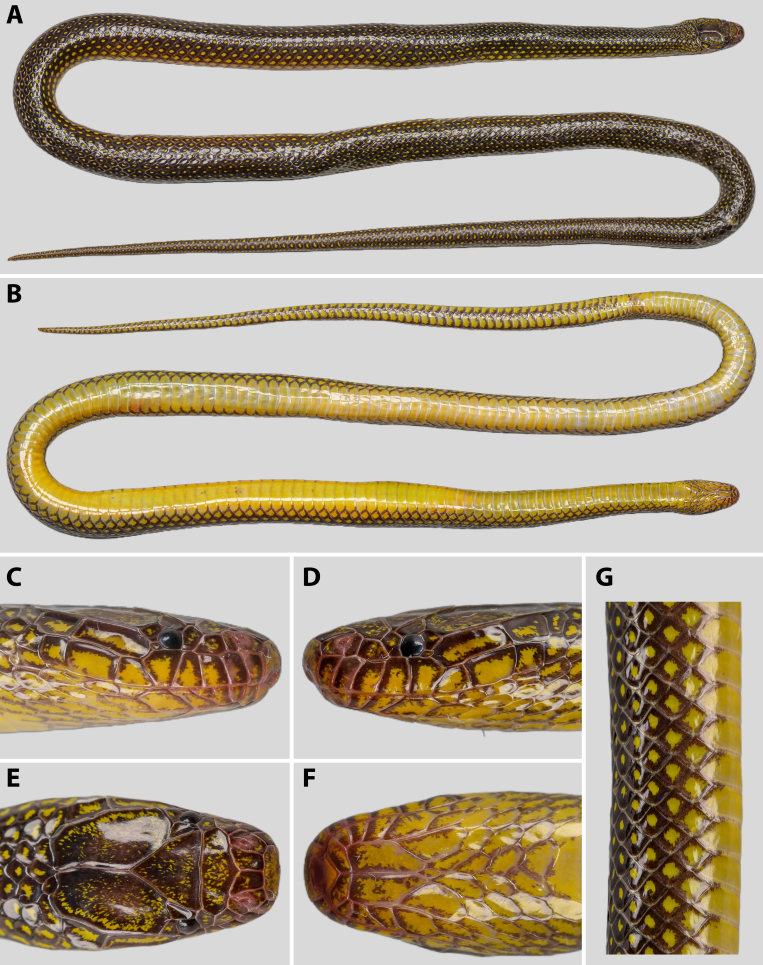
*Opisthotropis haihaensis* prior to preservation (specimen ANU 20240076, male): **A** dorsal view of whole body; **B** ventral view of whole body; **C** dorsal view of head; **D** ventral view of head; **E** right lateral view of head; **F** left lateral view of head; **G** close-up of mid-body dorsal scales. Photographs by T.R. Zhang.

**Coloration in life (Figs 1, 2).** Scales on dorsum of head glossy black with scattered yellow flecking; ventral of head yellow with brownish black mottling on the margins of each scale; upper lip yellow with brownish-black anterior and lateral margins on each supralabial; dorsum of body and tail glossy black with iridescence above, with single bright yellow spot on each scale, yellow spots becoming larger on sides of body; ventrals yellow with brownish black lateral margins; subcaudals yellow with brownish black anterior and lateral margins.

**Coloration in preservation.** Coloration and pigmentation patterns of newly preserved specimens (with 75% ethanol) significantly faded, bright yellow spots on dorsals and scattered yellow flecking on dorsum of head turn milky white, background color of ventrals fades to pale yellow. Gloss of scales remains distinct.

**Figure 3.**
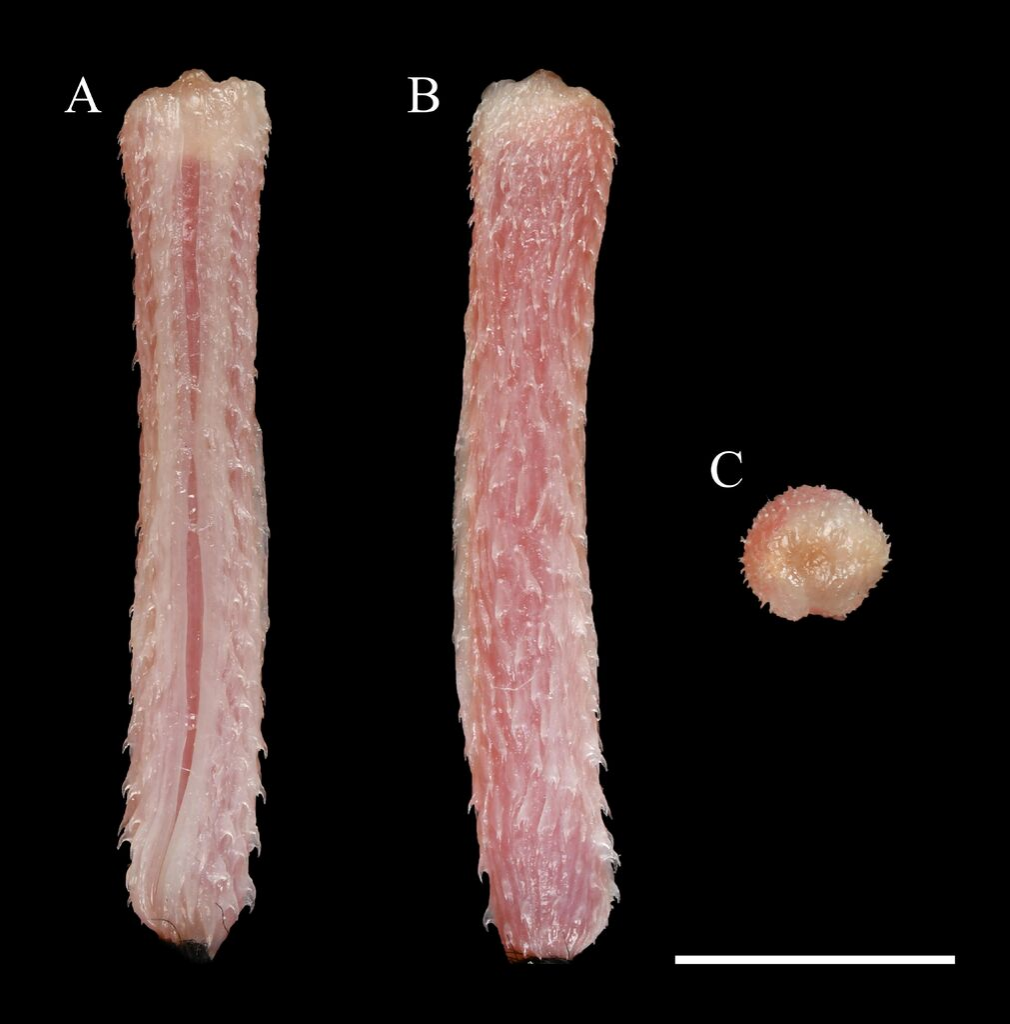
Hemipenis of *Opisthotropis haihaensis* (specimen ANU 20240076, male). **A** Sulcal view of the fully everted organ; **B** Lateral view showing overall shape and spine distribution; **C** Apical view of the hemipenial lobes. Photographs by J.J. Huang. Scale bar = 5 mm.

**Hemipenis** (Fig. 3). Fully everted hemipenis is relatively long, measuring 15.71 mm in length and 2.52 mm in width; slender, single, cylindrical, slightly clavate apically. Organ entirely spinose, with spines relatively uniform in size and distribution; spines slightly sparser in the proximal region; no basal nude area present. Basal hook absent. Sulcus spermaticus unforked, centripetal, extending straight to the apex; sulcus lips well developed, broad, and smooth along the entire length. When retracted, the hemipenis reaches the level of the 31^st^ subcaudal scale.

#### Description of additional female specimen ANU 20240077

**Measurements and scalation.** Body slender and cylindrical (TL 485.0 mm); head short and broad (HL/HW 1.76), dorsally depressed, barely distinct from neck; snout moderate (SnL/SnW 0.60); eye small, (ED/SoL 0.81); pupil round; interorbital distance large (IOD/HW 0.60); minute granular asperities absent on head scales; tail relatively long, tapering posteriorly (TaL 94.9 mm, TaL/TL 0.20); nostril oval-shaped, locating in the upper middle part of nasal, directed upwards.

Dorsal scales in 15 rows throughout the body; the outermost rows of dorsal scales slightly enlarged, while the rest ones homogeneous in size, smooth throughout; vertebral scales not enlarged; preventrals 1, ventrals 165; cloacal plate divided; subcaudals 71, paired, with single terminal rigid tip, smooth entirely.

Rostral crescent-shaped, wider than high, visible from above; nasals large, wider than high, divided below nostril by a distinct furrow; nasals in contact with the first two supralabials, rostral, internasal, prefrontal and loreal; internasals coalesced into one, pentagonal, wider than long, concaved inward posteriorly, in contact with rostral, nasal, and prefrontal; prefrontal single, much broader than long, anteriorly pointed, in contact with internasals, nasals, loreals, preoculars, and frontal; frontal single, pentagonal or near equilateral triangle, almost equal in width and length, tapering posteriorly, equal to its distance from tip of snout; parietals nearly 1.8 times as long as wide, parietal sutures nearly equal to frontal in length; 1/1 supraocular, right supraocular small and partially coalesced with upper postocular, distinctly longer than wide, not in contact with prefrontal; 1/1 loreal, pentagonal, wider than high, not entering orbit, surrounded by second and third supralabials, nasal, prefrontal and preocular; 1/1 preocular, large, hexagonal, higher than wide, reaching frontal; subocular absent; 2/2 postocular, upper pair in almost equal size of the lower pair; 7/7 supralabials, 1^st^–5^th^ higher than wide, 1^st^ and 2^nd^ partially coalesced on left lateral, only 4^th^ entering orbit on left lateral, 4^th^ and 5^th^ entering orbit on right lateral, 7^th^ largest; 1+1/1+1 temporals, anterior one very long and narrow, in broad contact with 5^th^–7^th^ supralabials and parietals, posterior one more strongly developed; infralabials 7/8, first pair in contact behind small mental, 1^st^–4^th^/1^st^–5^th^ in contact with anterior chin shields, 5^th^ infralabial largest; anterior chin shields longer than posterior ones, posterior chin shields do not contact with each other.

**Dentition.** Maxillary teeth 22, subequal, densely set, without diastema.

**Figure 4.**
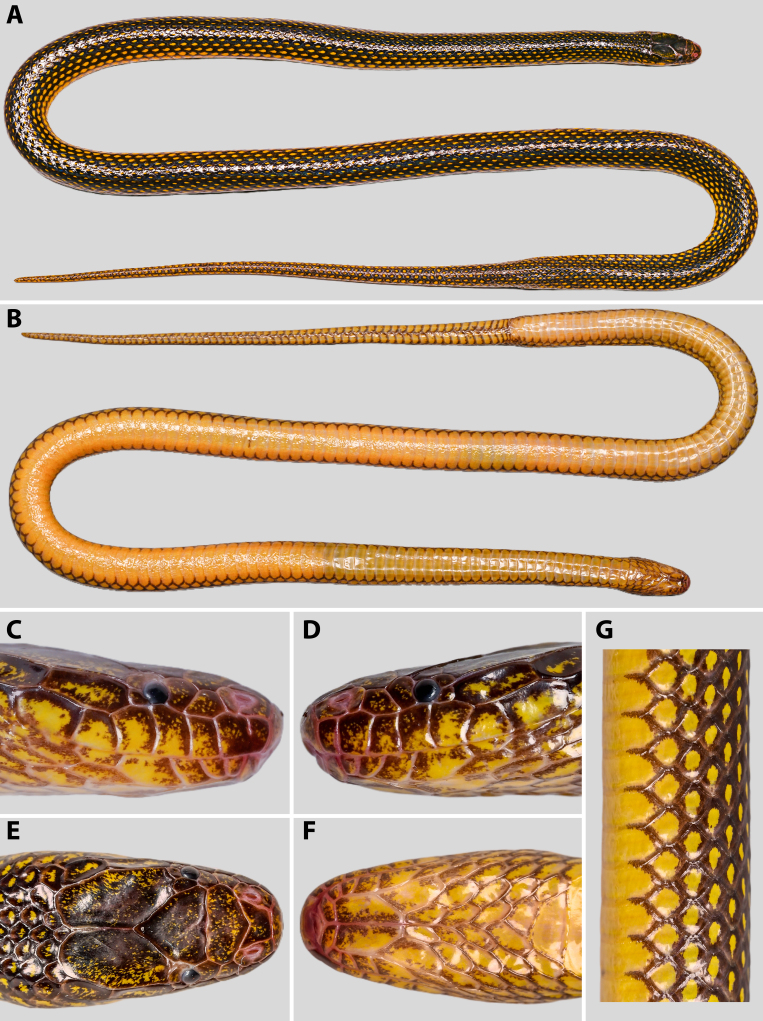
*Opisthotropis haihaensis* prior to preservation (specimen ANU 20240077, female): **A** dorsal view of whole body; **B** ventral view of whole body; **C** dorsal view of head; **D** ventral view of head; **E** right lateral view of head; **F** left lateral view of head; **G** close-up of midbody dorsal scales. Photographs by T.R. Zhang.

**Coloration in life and preservation** (Fig. 4). Similar to specimen ANU 20240076.

#### Variation.

The newly collected specimens conform to both the holotype (IEBR A.2016.34) from Vietnam and specimen SYS r000537 from China in general morphology, but exhibit the following differences (Tables 2, 3): shorter total and snout-vent lengths: TL 428.0–485.0 mm, SVL 314.1–387.1 mm (vs. TL 500.2–509.0 mm, SVL 391.3–396.0 mm); lower number of supralabials: 7 (vs. 8); broader range of tail length: TaL 113.9 mm and TaL/TL 0.27 in the male; TaL 94.9 mm and TaL/TL 0.20 in the female (vs. TaL 108.9–113.0 mm and TaL/TL 0.22 in females); wider range of subcaudal counts: 87 in the male and 71 in the female (vs. 75–79 in females); variation in supralabials entering the orbit: 4^th^ and 5^th^ or only 4^th^ or 5^th^ (vs. consistently 4^th^ and 5^th^).

#### Revised diagnosis.

*Opisthotropis haihaensis* can be distinguished from all congeners by the following combination of characters: (1) adult females reach a total length (TL) of 485–509 mm, and the only examined adult male measures 428 mm in TL; (2) the tail is relatively long, with a TaL/TL ratio of 0.20–0.22 in females and 0.27 in the male; (3) the species exhibits 7–8 supralabials, with the 4^th^ and 5^th^ or either the 4^th^ or 5^th^, entering the orbit; (4) the 6^th^ supralabial is not elongated and is nearly or less than half the length of the anterior temporal; (5) preocular one, postoculars one or two; (6) the internasals do not contact the loreal, and the prefrontals are separated from the supraoculars, while the frontal is in contact with the preocular; (7) the temporal formula is either 1 or 1+1; (8) the species has 164–169 ventrals and 87 subcaudals in the male and 71–79 in females; (9) dorsal scales in 15–15–15 rows and are smooth on the body, with the tail scales either smooth or indistinctly keeled; (10) the chin shields are yellow with brownish black mottling, and the body and tail dorsum is dark, each dorsal scale bearing a distinct pale spot; (11) the maxillary teeth number 22–24, are subequal in size, closely spaced, and lack a diastema; (12) the hemipenis is single, cylindrical, fully spinose throughout, and lacks a basal hook.

#### Distribution

**Figure 5.**
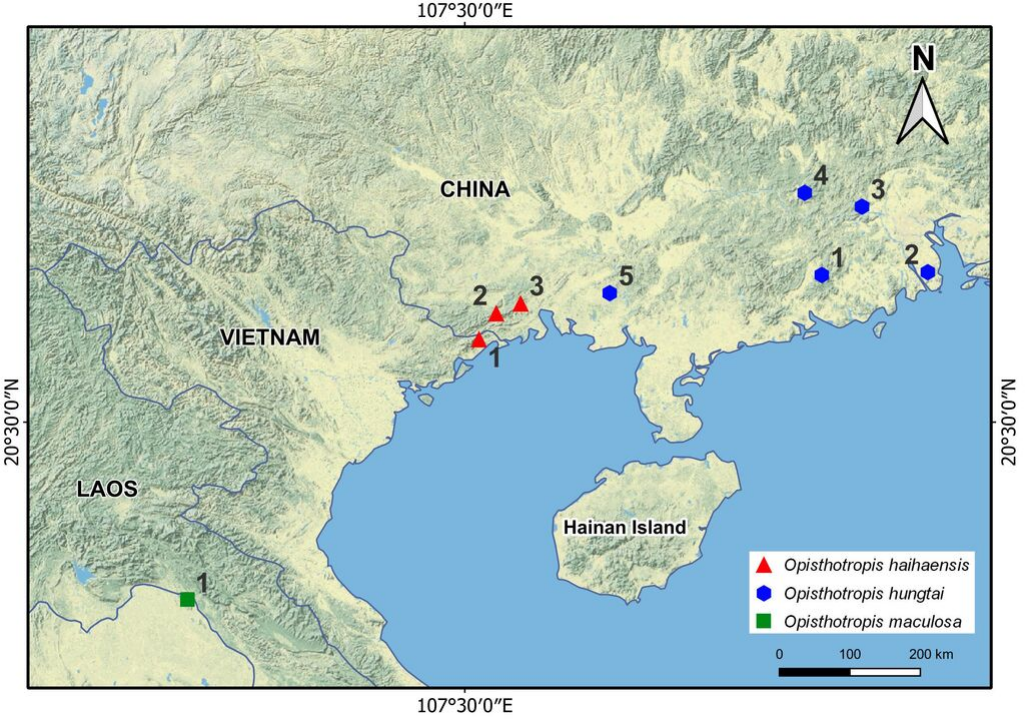
Distribution ranges of *Opisthotropis maculosa* complex species. Numbers correspond to locality records (see Table 4 for locality details).

*Opisthotropis haihaensis* is currently known from three localities in northern Vietnam and southern China (Fig. 5, Table 4): (1) the type locality in Quang Son Commune, Hai Ha District, Quang Ninh Province, Vietnam; (2) Shiwandashan National Nature Reserve, Shangsi County, Fangchenggang City, Guangxi ZAR, China; and (3) Qinbei District, Qinzhou City, Guangxi ZAR, China.

#### Natural history notes.

*Opisthotropis haihaensis* inhabits small, slow-flowing mountain streams within south subtropical evergreen broad-leaved forests at elevations of 500–950 m above sea level in northern Vietnam and southern China. These streams are consistently clear and rocky, with occasional deposits of humus on the streambed. In captivity, both examined specimens exhibited a strong predatory response to earthworms (*Pheretima guillelmi* Michaelsen) measuring 90–150 mm in length, suggesting feeding behavior similar to that observed in other congeners.

## Identification Keys

### Revised key to the 13 species of *Opisthotropis* occurring in China

**Table:** 

1	Dorsal scales in 19 or 23 at midbody	2
–	Dorsal scales in 15 or 17 rows at midbody	4
2	Dorsal scales in 19 rows at midbody	3
–	Dorsal scales in 23 rows at midbody	*O. laui*
3	Supralabials 13–16, with several posterior ones divided horizontally; dorsal scales strongly keeled throughout; brown above with black longitudinal stripes on body and tail	*O. kuatunensis*
–	Supralabials 9–10, not divided horizontally; dorsal scales weakly keeled on neck, strongly keeled on body and tail; olive-green above with fine black mesh pattern	*O. shenzhenensis*
4	Dorsal scales in 15 rows at midbody	5
–	Dorsal scales in 17 rows at midbody	8
5	Dorsal scale row formula 17–15–15; body with narrow pale yellow crossbars	*O. guangxiensis*
–	Dorsal scale row formula 15–15–15; body without pale yellow crossbars	6
6	Dorsum uniformly dark; dorsal scales pale-edged posteriorly	*O. jacobi*
–	Dorsum olive-brown, each dorsal scale with a single yellow spot	7
7	The 6^th^ supralabial is not elongated, approximately equal to or shorter than half the length of the anterior temporal	*O. haihaensis*
–	The 6^th^ supralabial is distinctly elongated and nearly equal in length to the anterior temporal	*O. hungtai*
8	A lateral black stripe separating pale venter from dark dorsum; loreal not entering orbit	*O. lateralis*
–	No such lateral stripe; loreal entering orbit or not	9
9	Loreal not entering orbit, 1.4–1.7× as long as deep; last supralabial longest	*O. maxwelli*
–	Loreal entering orbit or not, more than 1.7× as long as deep; last supralabial smaller than preceding one	10
10	Loreal in contact with second supralabial; tail length 15–20% of total length; dorsum without yellow longitudinal stripes or crossbars	*O. andersonii*
–	Loreal not contacting second supralabial; tail length 20–23% of total length; dorsum with yellow longitudinal stripes or crossbars	11
11	Body size small, total length shorter than 419 mm; maxillary teeth ≤ 25	*O. latouchii*
–	Body size moderate or large, total length longer than 419 mm; maxillary teeth ≥ 25	12
12	Maxillary teeth 25–28; anterior temporals elongated, maximum anterior temporal length / depth ratio 2.63–3.63; dorsum dark olive with yellow crossbars	*O. cheni*
–	Maxillary teeth 28–30; anterior temporals short, maximum anterior temporal length / depth ratio 1.74–2.04; dorsum with longitudinal yellow stripes	*O. zhaoermii*

Modified from Zhao (2006), Yang et al. (2011), Ren et al. (2017), Ren (2019) and this study.

## Analysis

**Figure 6.**
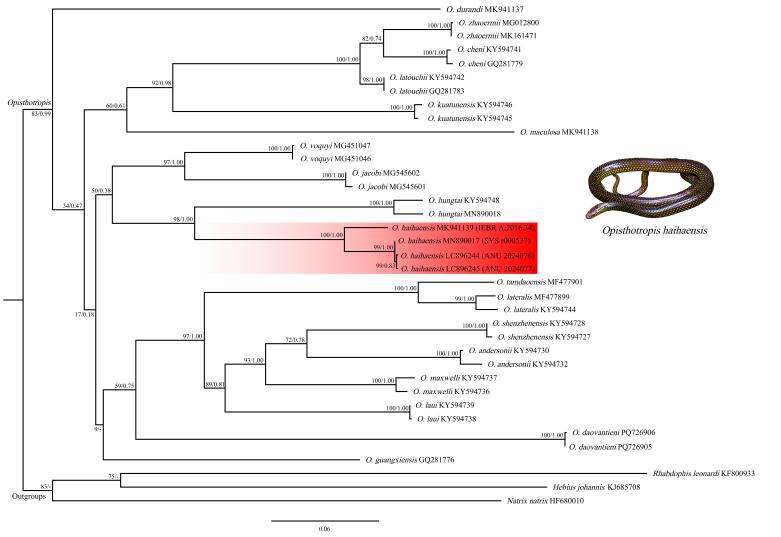
ML phylogenetic tree estimated from Cyt *b* sequences depicting phylogenetic relationships of *Opisthotropis* (numbers above branches are BSP/BPP).

In this study, 1 060 base pairs (bp) of Cyt *b* sequences were successfully obtained for alignment, including two newly generated sequences of *O. haihaensis*. Both ML and BI analyses yielded highly congruent topologies (Fig. 6), consistent with findings from previous studies (Ren et al. 2017, Wang et al. 2017, Ziegler et al. 2019, Wang et al. 2020).

The genus *Opisthotropis* was recovered as a monophyletic group within the scope of the current sampling (BSP = 83, BPP = 0.99). All *O. haihaensis* individuals clustered within a distinct and well-supported clade (BSP = 100, BPP = 1.00), nested within the *Opisthotropis* clade. Infraspecific uncorrected p-distances among the *O. haihaensis* specimens were 0.2% –4.5% (Suppl. material 1). The *p*-distances between *O. haihaensis* and all analyzed *Opisthotropis* species varied from 13.3%–13.4% (for *O. hungtai*) to 18.0%–18.3% (for *O. daovantieni* Orlov, Darevsky & Murphy). The following is a detailed description of the additional specimens of *O. haihaensis*.

## Discussion

Wang et al. (2020) considered the number of supralabials to be a key diagnostic character distinguishing *Opisthotropis haihaensis* from *O. hungtai*, with the former bearing eight supralabials and the latter seven. However, our findings indicate that supralabial counts may vary depending on the position of the oral fissure relative to the posterior margin of the seventh supralabial, potentially leading to misinterpretation. We also re-checked the supralabial counts of *O. haihaensis* specimen SYS r000537 and found it actually has 7 supralabials (8 in Yang et al. (2011), Wang et al. (2020)). To minimize diagnostic ambiguity caused by variable supralabial counts, we propose an alternative diagnostic character: in *O. haihaensis*, the 6^th^ supralabial is not elongated and is approximately equal to or shorter than half the length of the anterior temporal, whereas in *O. hungtai*, the 6^th^ supralabial is distinctly elongated and nearly equal in length to the anterior temporal.

Among the 25 currently recognized species of *Opisthotropis*, hemipenial morphology has been described in only 13 species (see Table 5) and several of these descriptions remain brief or lack sufficient detail (Pope 1935, Zhang et al. 1984, Stuart and Chuaynkern 2007, Yang et al. 2011, Ziegler et al. 2018, Ren 2019, Gao et al. 2024). Based on the most recent summaries by Gao et al. (2024), the general hemipenial morphology of *Opisthotropis* is characterized by the following traits: relatively short and stout hemipenis, shallowly bilobed in a “Y” shape, noncalyculate and noncapitate (except in *O. alcalai* and *O. maculosa*); uniform coverage with small spines or spinules; a single enlarged basal hook on the proximal region of the truncus (except in *O. maculosa*); and a single, undivided, centripetal sulcus spermaticus extending to the base or distal portion of the inner right lobe (except in *O. daovantieni*). Within this framework, *Opisthotropis haihaensis* and *O. maculosa* exhibit unique hemipenial morphology in possessing a single, cylindrical hemipenis entirely spinose and lacking a basal hook distinguishing them from other congeners. Presence or absence of the basal hook and morphology of sulcus serve as significant taxonomic diagnostic criteria, as they could directly influence reproductive behavior and reflect adaptive evolution in reproductive strategies. The hemipenis of *Opisthotropis hungtai*, the species most closely related to *O. haihaensis*, remains undescribed. Yang et al. (2011) briefly noted that the hemipenis of specimen SYS r0538 is cylindrical and extends to the level of the 12^th^ subcaudal and provided an image of KFBG 2002.01. However, the structure shown does not appear to be fully everted, rendering any morphological inference inconclusive. Detailed documentation of hemipenes in *Opisthotropis hungtai* and other poorly characterized or undocumented species is essential for evaluating patterns of hemipenial variation and their potential phylogenetic significance.

*Opisthotropis haihaensis* is currently known from only four specimens collected at three localities in northern Vietnam and southern China. It inhabits clear, slow-flowing mountain streams within evergreen broad-leaved forests habitats that are increasingly threatened by deforestation and other anthropogenic disturbances. Given its narrow known distribution and extremely limited number of recorded specimens, we recommend a provisional IUCN Red List status of Near Threatened (NT). This classification is precautionary, pending further field surveys and population assessments, and reflects the urgent need for more comprehensive ecological and conservation data.

## Supplementary Material

XML Treatment for Opisthotropis haihaensis

4404A7D8-678D-593B-BF45-66B77B6AF847 10.3897/BDJ.13.e167521.suppl1 Supplementary material 1 Uncorrected pairwise sequence divergence Data type Phylogenetic Brief description Uncorrected pairwise sequence divergence (%) among Cyt *b* mtDNA gene sequences of *Opisthotropis* species. File: oo_1385360.doc https://binary.pensoft.net/file/1385360 Tierui Zhang, Jinlong Ren, Maoliang Li, Yuhao Xu, Xinge Wang, Nikolay A. Poyarkov, Tan Van Nguyen, Song Huang

## Figures and Tables

**Table 1.** Information of samples and sequences used in molecular analysis. Notes: Is = Island; NP = National Park; NR = Nature Reserve; WS = Wildlife Sanctuary; * = DDBJ Accession Number.

**Species**	**Voucher No.**	**NCBI/DDBJ acc. No.**	**Locality**
*Opisthotropis andersonii*	SYS r001020	KY594732	China: Mt. Wutong, Shenzhen, Guangdong
*Opisthotropis andersonii*	SYS r001423	KY594730	China: Tai Tam, Hong Kong
*Opisthotropis cheni*	YBU 071040	GQ281779	China: Guangdong
*Opisthotropis cheni*	SYS r001422	KY594741	China: Shimentai NR, Yingde, Guangdong
*Opisthotropis daovantieni*	CIB 109024	PQ726905	Vietnam: K’Bang, Gia Lai
*Opisthotropis daovantieni*	VNMN 2019.01	PQ726906	Vietnam: K’Bang, Gia Lai
*Opisthotropis durandi*	NCSM 80739	MK941137	Laos: Phongsaly
*Opisthotropis guangxiensis*	GP 746	GQ281776	China: Guangxi
*Opisthotropis haihaensis*	IEBR A.2016.34	MK941139	Vietnam: Hai Ha, Quang Ninh
*Opisthotropis haihaensis*	SYS r000537	MN890017	China: Shiwandashan NR, Guangxi
*Opisthotropis haihaensis*	ANU 20240076	LC896244*	China: Qinbei, Qinzhou, Guangxi
*Opisthotropis haihaensis*	ANU 20240077	LC896245*	China: Qinbei, Qinzhou, Guangxi
*Opisthotropis hungtai*	SYS r000538	MN890018	China: Mt. Wuhuang, Guangxi
*Opisthotropis hungtai*	SYS r000946	KY594748	China: Heishiding NR, Fengkai, Guangdong
*Opisthotropis jacobi*	IEBR 4329	MG545601	Vietnam: Tam Dao NP, Vinh Phuc
*Opisthotropis jacobi*	ZFMK 100818	MG545602	Vietnam: Tam Dao NP, Vinh Phuc
*Opisthotropis kuatunensis*	SYS r000998	KY594745	China: Qixiling NR, Yongxin, Jiangxi
*Opisthotropis kuatunensis*	SYS r001008	KY594746	China: Wulong, Shanghang, Fujian
*Opisthotropis laterialis*	SYS r001080	KY594744	China: Mt. Wutong, Shenzhen, Guangdong
*Opisthotropis laterialis*	ZFMK 100806	MF477899	Vietnam: Tay Yen Tu NR, Bac Giang
*Opisthotropis latouchii*	GP 647	GQ281783	China: Fujian
*Opisthotropis latouchii*	SYS r000670	KY594742	China: Guadun, Wuyishan, Fujian
*Opisthotropis laui*	SYS r001161	KY594738	China: Shangchuan Is, Taishan, Guangdong
*Opisthotropis laui*	SYS r001170	KY594739	China: Shangchuan Is, Taishan, Guangdong
*Opisthotropis maculosa*	FMNH 265798	MK941138	Thailand: Phu Wua WS, Nong Khai
*Opisthotropis maxwelli*	SYS r000841	KY594736	China: Nan’ao Is, Guangdong
*Opisthotropis maxwelli*	SYS r001053	KY594737	China: Huboliao NR, Nanjing, Fujian
*Opisthotropis shenzhenensis*	SYS r001018	KY594727	China: Mt. Wutong, Shenzhen, Guangdong
*Opisthotropis shenzhenensis*	SYS r001021	KY594728	China: Sanzhoutian, Shenzhen, Guangdong
*Opisthotropis tamdaoensis*	IEBR A.2016.33	MF477901	Vietnam: Tam Dao NP, Vinh Phuc
*Opisthotropis voquyi*	IEBR 4327	MG451046	Vietnam: Tay Yen Tu NR, Bac Giang
*Opisthotropis voquyi*	VNMN 06315	MG451047	Vietnam: Tay Yen Tu NR, Bac Giang
*Opisthotropis zhaoermii*	CIB 109999	MG012800	China: Guzhang, Hunan
*Opisthotropis zhaoermii*	GZNU 320074	MK161471	China: Leishan, Guizhou
*Hebius johannis*	GP 897	KJ685708	China: Yunnan
*Natrix natrix*	MTD T 9269	HF680010	Denmark: Sønder Borup, Zealand
*Rhabdophis leonardi*	SCUM 090009	KF800933	China: Panzhihua, Sichuan

**Table 2.** Morphological comparisons among newly collected specimens and previously reported specimens of *Opisthotropis haihaensis*. Notes: * = Holotype.

**Voucher No.**	**ANU 20240076**	**ANU 20240077**	IEBR A.2016.34*	**SYS r000537**
**Sex**	M	F	F	F
**TaL (mm)**	113.9	94.9	113.0	108.9
**TL (mm)**	428.0	482.1	509.0	500.2
**TaL/TL**	0.27	0.20	0.22	0.22
**PtO**	2/2	2/2	1/1	2/2
**SL**	7/7	7/7	8/8	7/7
**SL-orbit**	5^th^/4^th^–5^th^	4^th^/4^th^–5^th^	4^th^–5^th^/4^th^–5^th^	4^th^–5^th^/4^th^–5^th^
**IL**	8/8	7/8	8/8	7/7
**IL-aCS**	1^st^–5^th^/1^st^–5^th^	1^st^–4^th^/1^st^–5^th^	1^st^–4^th^/1^st^–5^th^	1^st^–5^th^/1^st^–4^th^
**PrF-SpO**	No	No	No	No
**TEM**	1/1	1+1/1+1	1+1/1+1	1+1/1+1
**MT**	22	22	24	22
**DSR**	15-15-15	15-15-15	15-15-15	15-15-15
**VEN**	168	165	169	164
**SC**	87	71	79	75
**CP**	divided	divided	divided	divided
**DST**	smooth	smooth	smooth	indistinctly keeled
**Source**	This study	This study	Nguyen et al. (2018), Ziegler et al. (2019)	Yang et al. (2011), Wang et al. (2020), This study

**Table 3.** Morphological comparisons among three recognized species included in *Opisthotropis maculosa* complex species. Notes: “?” indicates missing data, M = males, F = females.

**Species**	***O* . *haihaensis***	***O. hungtai***	***O. maculosa***
**Number specimens**	N = 4 (M = 1, F = 3)	N = 8 (M = 4, F = 4)	N = 1 (M = 1)
**TaL**	94.9–113.9	81.2–120.7	110
**TL**	428.0–509.0	366.0–511.0	520
**TaL/TL**	0.20–0.27	0.19–0.26	0.21
**PrO**	1	1	1
**PtO**	1 or 2	1	2
**SL**	7–8	7	8
**SL-orbit**	4^th^–5^th^ or 4^th^ or 5^th^	4^th^–5^th^	4^th^
**IL**	7–8	7–9	8
**IL-aCS**	1^st^–4^th^ or 1^st^–5^th^	1^st^–4^th^	1^st^–5^th^
**PrF-SpO**	No	No	Yes
**IN-L**	No	No	No
**PrO-F**	Yes	Yes	No
**TEM**	1 or 1+1	1+1	1+1
**MT**	22–24	16–18	?
**DSR**	15-15-15	15-15-15	15-15-15
**VEN**	164–169	168–189	182
**SC**	71–87	69–98	67
**CP**	divided	divided	divided
**DSB**	smooth	smooth	smooth
**DST**	smooth or indistinctly keeled	weakly keeled	smooth
**Hemipenis**	single cylindrical, spinose throughout	“cylindrical”	single cylindrical, basal area naked
**Source**	Yang et al. (2011), Nguyen et al. (2018), Ziegler et al. (2019), Wang et al. (2020), this study	Yang et al. (2011), Wang et al. (2020)	Stuart and Chuaynkern (2007)

**Table 4.** Localities of *Opisthotropis maculosa* complex specimens shown in Figure 5. Symbols: (1) = locality number corresponding to the map in Fig. 6; (2) = verified by morphological data (yes/no); (3) = verified by molecular data (yes/no). Abbreviations: NR = Nature Reserve; WS = Wildlife Sanctuary.

**(1)**	**(2)**	**(3)**	**Location**	**Sources**
***Opisthotropis haihaensis***				
1	yes	yes	Tai Chi, Quang Son, Hai Ha, Quang Ninh, Vietnam (type locality)	Nguyen et al. (2018), Ziegler et al. (2019)
2	yes	yes	Shiwandashan NR, Fangchenggang, Guangxi, China	Yang et al. (2011), Wang et al. (2020)
3	yes	yes	Qinbei, Qinzhou, Guangxi, China	this study
***Opisthotropis hungtai***				
1	yes	yes	Heishiding NR, Guangdong, China (type locality)	Wang et al. (2020)
2	yes	no	Wuguishan, Zhongshan, Guangdong, China	https://www.inaturalist.org/observations/ 206074243
3	yes	no	Dawuling Forestry Station, Guangdong, China	Wang et al. (2020)
4	yes	no	Zhaoqing, Guangdong, China	https://www.inaturalist.org/observations/26850282
5	yes	yes	Mt. Wuhuang, Guangxi, China	Wang et al. (2020)
***Opisthotropis maculosa***				
1	yes	yes	Phu Wua WS, Boong Klar, Nong Khai, Thailand	Stuart and Chuaynkern (2007)

**Table 5.** Documented hemipenial morphology of *Opisthotropis* species. Notes: Relative length of retracted hemipenis was defined by the subcaudal it can reach at the distalmost; “?” indicates missing data.

**Species**	**Shape**	**Ornamentation**	**Enlarged basal hook**	***Sulcus spermaticus***	**Relative length of retracted hemipenis**	**Source**
***O. alcalai***	Single	Uniformly spinose throughout	1	Undivided, extends to apical tip	11^th^	Brown and Leviton (1961)
***O. andersonii***	?	Beset with spines proximally and papilla-like processes distally	1	?	?	Pope (1935)
***O. daovantieni***	Bilobed, lobes well- developed	Spinose throughout, spines approximately homogeneous in size, proximal fourth sparser, without basal naked area	1	Bifurcate, centripetal, reaching apex of each lobe	17^th^	Gao et al. (2024)
***O. guangxiensis***	Bilobed, short and thin, slightly stouter at the proximal half	Spinose throughout, proximal third sparser.	1	Undivided, reaching apex of the right lobe	?	Ren (2019), Gao et al. (2024)
***O. haihaensis***	Single, cylindrical, slightly clavate apically	Spinose throughout, spines approximately homogeneous in size, proximal sparser slightly, without basal naked area.	Absent	Undivided, centripetal, reaching apex of hemipenis	31^st^	This study
***O. hungtai***	Cylindrical	?	?	?	12^th^	Yang et al. (2011)
***O. jacobi***	Bilobed, lobes short, inwards curved	Covered with small spines, curved backwards. The terminal area of the hemipenis between the lobes without spines. Upper truncus of hemipenes with ring of enlarged spines	1	Undivided, stretching to the slightly smaller lobe only.	?	Ziegler et al. (2018)
***O. kuatunensis***	Slightly divided at the tip	Spinous, spines are set in longitudinal rows and connected by fleshy ridges, recurved proximally and gradually decrease in distal.	1	Undivided	10^th^	Pope (1935)
**O. lateralis**	?	Beset with spines proximally and papilla-like processes distally	2	?	?	Pope (1935)
***O. latouchii***	Bilobed, slightly forked at the tip	Beset with hook-shaped spines proximally and papilla-like processes distally	1	Undivided, reaching apex of the right lobe	6^th^–8^th^	Pope (1935), Zhang et al. (1984), Ren (2019)
***O. laui***	Bilobed, slightly elongated	Beset with hook-shaped spines proximally and papilla-like processes distally	1	?	?	Ren (2019)
***O. maculosa***	Single, cylindrical	Basal area naked, remainder of hemipenis uniformly ornamented with spines	Absent	Undivided, centripetal, reaching apex of hemipenis	12^th^	Stuart and Chuaynkern (2007)
***O. voquyi***	Bilobed, lobes short, inwards curved	Covered with small spines, curved backwards. The terminal area of the hemipenes between the lobes without spines. Upper truncus of hemipenes with ring of enlarged spines.	1	Undivided, stretching to the slightly smaller lobe only.	?	Ziegler et al. (2018)
***O. zhaoermii***	Bilobed, right lobe shorter than the left lobe	Covered with small spines. The terminal area of the hemipenis between the lobes and basal area naked. Spines around basal hook moderately enlarged, spines on the remainder of hemipenis approximately homogeneous in size	1	Undivided, reaching apex of the right lobe	?	Gao et al. (2024)
