# Supplementary material for: First description of the male and hemipenial morphology of Opisthotropis haihaensis Ziegler et al., 2019 (Serpentes, Natricidae), with updated diagnosis and distribution
Source: Biodivers Data J. 2025 Nov 20;13:e167521. doi: 10.3897/BDJ.13.e167521 (PMC12661333; doi:10.3897/BDJ.13.e167521)
Supplement: Supplementary material 1 — Uncorrected pairwise sequence divergence [file bdj-13-e167521-s001.doc]

**Table S3.** Uncorrected pairwise sequence divergence (%) among Cyt *b* mtDNA gene sequences of *Opisthotropis* species.

| **#** | **Species** | **Voucher No.** | **1** | **2** | **3** | **4** | **5** | **6** | **7** | **8** | **9** |
| --- | --- | --- | --- | --- | --- | --- | --- | --- | --- | --- | --- |
| **1** | ***O*. *haihaensis*** | ANU 20240076 |  |  |  |  |  |  |  |  |  |
| **2** | ***O*. *haihaensis*** | ANU 20240077 | 0.2 |  |  |  |  |  |  |  |  |
| **3** | ***O*. *haihaensis*** | IEBR A.2016.34 | 4.5 | 4.5 |  |  |  |  |  |  |  |
| **4** | ***O*. *haihaensis*** | SYS r000537 | 0.2 | 0.2 | 4.4 |  |  |  |  |  |  |
| **5** | ***O*. *andersonii*** | SYS r001020 | 16.0 | 16.1 | 16.4 | 16.0 |  |  |  |  |  |
| **6** | ***O*. *cheni*** | SYS r001422 | 15.4 | 15.4 | 15.9 | 15.6 | 17.3 |  |  |  |  |
| **7** | ***O*. *daovantieni*** | CIB109024 | 18.3 | 18.5 | 18.0 | 18.3 | 15.9 | 17.5 |  |  |  |
| **8** | ***O*. *durandi*** | NCSM 80739 | 17.2 | 17.3 | 17.0 | 17.2 | 17.3 | 18.2 | 17.5 |  |  |
| **9** | ***O*. *guangxiensis*** | GP 746 | 14.4 | 14.4 | 14.5 | 14.5 | 14.9 | 16.3 | 17.6 | 15.6 |  |
| **10** | ***O*. *hungtai*** | SYS r000538 | 13.4 | 13.4 | 13.3 | 13.4 | 16.2 | 17.2 | 18.4 | 17.3 | 15.6 |
| **11** | ***O*. *jacobi*** | ZFMK 100818 | 13.9 | 14.0 | 14.3 | 13.9 | 16.1 | 14.7 | 16.5 | 15.8 | 13.6 |
| **12** | ***O*. *kuatunensis*** | SYS r001008 | 16.1 | 16.2 | 16.4 | 16.1 | 16.6 | 14.3 | 17.8 | 17.3 | 15.9 |
| **13** | ***O*. *lateralis*** | SYS r001080 | 16.1 | 16.2 | 16.0 | 16.1 | 14.6 | 15.8 | 18.9 | 17.2 | 16.7 |
| **14** | ***O*. *latouchii*** | SYS r000670 | 15.3 | 16.1 | 16.0 | 15.3 | 15.9 | 5.0 | 16.9 | 17.4 | 15.5 |
| **15** | ***O*. *laui*** | SYS r001170 | 16.1 | 16.1 | 15.4 | 16.1 | 12.2 | 16.7 | 17.9 | 17.9 | 16.4 |
| **16** | ***O*. *maculosa*** | FMNH 265798 | 16.2 | 16.2 | 17.3 | 16.2 | 18.5 | 17.7 | 18.7 | 16.8 | 15.9 |
| **17** | ***O*. *maxwelli*** | SYS r001053 | 16.1 | 16.1 | 15.3 | 15.9 | 12.4 | 16.3 | 18.0 | 17.8 | 15.8 |
| **18** | ***O*. *shenzhenensis*** | SYS r001021 | 17.6 | 17.5 | 17.3 | 17.5 | 11.5 | 17.1 | 18.2 | 17.9 | 16.4 |
| **19** | ***O*. *tamdaoensis*** | IEBR A.2016.33 | 16.3 | 16.4 | 16.3 | 16.3 | 14.9 | 16.7 | 19.1 | 16.8 | 17.3 |
| **20** | ***O*. *voquyi*** | VNMN 06315 | 13.4 | 13.4 | 13.4 | 13.4 | 14.5 | 14.3 | 16.8 | 15.3 | 13.5 |
| **21** | ***O*. *zhaoermii*** | CIB 109999 | 15.3 | 15.5 | 15.8 | 15.5 | 15.9 | 5.6 | 17.6 | 17.8 | 15.9 |

**Table S3. (Continue)** Uncorrected pairwise sequence divergence (%) among Cyt *b* mtDNA gene sequences of *Opisthotropis* species.

| **#** | **Species** | **Voucher No.** | **10** | **11** | **12** | **13** | **14** | **15** | **16** | **17** | **18** | **19** | **20** |
| --- | --- | --- | --- | --- | --- | --- | --- | --- | --- | --- | --- | --- | --- |
| **11** | ***O*. *jacobi*** | ZFMK 100818 | 14.0 |  |  |  |  |  |  |  |  |  |  |
| **12** | ***O*. *kuatunensis*** | SYS r001008 | 15.6 | 15.2 |  |  |  |  |  |  |  |  |  |
| **13** | ***O*. *lateralis*** | SYS r001080 | 18.6 | 17.5 | 16.7 |  |  |  |  |  |  |  |  |
| **14** | ***O*. *latouchii*** | SYS r000670 | 16.6 | 14.7 | 14.0 | 15.9 |  |  |  |  |  |  |  |
| **15** | ***O*. *laui*** | SYS r001170 | 15.4 | 15.6 | 16.6 | 15.1 | 16.2 |  |  |  |  |  |  |
| **16** | ***O*. *maculosa*** | FMNH 265798 | 17.9 | 15.9 | 16.5 | 17.7 | 17.1 | 18.6 |  |  |  |  |  |
| **17** | ***O*. *maxwelli*** | SYS r001053 | 15.3 | 16.0 | 16.0 | 13.8 | 15.8 | 11.9 | 17.6 |  |  |  |  |
| **18** | ***O*. *shenzhenensis*** | SYS r001021 | 16.2 | 16.0 | 17.3 | 14.7 | 16.2 | 14.1 | 18.1 | 11.2 |  |  |  |
| **19** | ***O*. *tamdaoensis*** | IEBR A.2016.33 | 17.3 | 17.2 | 16.9 | 6.3 | 16.0 | 14.4 | 18.0 | 14.8 | 15.2 |  |  |
| **20** | ***O*. *voquyi*** | VNMN 06315 | 14.9 | 9.9 | 15.7 | 14.7 | 14.2 | 13.6 | 16.4 | 15.3 | 14.9 | 15.3 |  |
| **21** | ***O*. *zhaoermii*** | CIB 109999 | 17.0 | 14.2 | 14.7 | 16.1 | 5.1 | 16.5 | 17.5 | 15.8 | 15.9 | 16.4 | 14.5 |
